# Post-ICU discharge and outcome: rationale and methods of the The French and euRopean Outcome reGistry in Intensive Care Units (FROG-ICU) observational study

**DOI:** 10.1186/s12871-015-0129-2

**Published:** 2015-10-12

**Authors:** Alexandre Mebazaa, Maria Chiara Casadio, Elie Azoulay, Bertrand Guidet, Samir Jaber, Bruno Levy, Didier Payen, Eric Vicaut, Matthieu Resche-Rigon, Etienne Gayat

**Affiliations:** 1 Department of Anesthesiology and Intensive Care, Saint Louis – Lariboisière – Fernand Widal University Hospital, AP-HP, Paris, France; 2 INSERM UMR-S 942, Paris, France; 3 Department of Anesthesiology and Critical Care Medicine, Saint Louis – Lariboisière University Hospital, University Paris Diderot, UMR-S 942, INSERM, 2 rue Ambroise Paré, 75010 Paris, France; 4 DHU Neurovasculaire, Paris, France; 5 Medical intensive care, Saint Louis – Lariboisière – Fernand Widal University Hospital, AP-HP, Paris, France; 6 INSERM UMR-S 717, Paris, France; 7 Medical intensive care, Saint Antoine University Hospital, AP-HP, Paris, France; 8 INSERM UMR 1136, Paris, France; 9 University Paris VI, Paris, France; 10 Surgical Intensive Care, Saint Eloi University Hospital, Montpellier, France; 11 Medical intensive care, CHU Nancy - Hôpital Brabois Adultes, Vandœuvre-les-Nancy, France; 12 INSERM UMR-S 1160, Paris, France; 13 Unité de Recherche Clinique, Saint Louis – Lariboisière – Fernand Widal University Hospital, AP-HP, Paris, France; 14 Service de Biostatistique et d’Informatique Médicale, Saint Louis – Lariboisière – Fernand Widal University Hospital, AP-HP, Paris, France

**Keywords:** Intensive care unit, Outcome, Mortality, Biomarkers

## Abstract

**Background:**

Previous studies have demonstrated that ICU (intensive care unit) survivors have decreased long-term survival rates compared to the general population. However, knowledge about how to identify ICU survivors with higher risk of death and the adjustable factors associated with mortality is still lacking.

**Methods and Design:**

The FROG-ICU (the French and European Outcome Registry in Intensive Care Units) study is a prospective, observational, multicenter cohort study where ICU survivors are followed up to one year after ICU discharge. Beside one year survival, the study is designed to assess incidence and identifying risk factors for mortality over the year following discharge from the ICU. All consecutive patients admitted in ICU to the 28 participating centers during the study period will be included. Every subject will undergo an evaluation at admission, throughout the ICU stay and at ICU discharge. The global, especially cardiovascular, assessment of each subject will be performed through a complete clinical exam, instrumental tests (electrocardiogram, echocardiogram) and biological parameters. Blood and urine samples will be collected at admission and at discharge with the primary goal to assess effectiveness of routine and novel cardiovascular, inflammatory and renal biomarkers, with potential interest in risk stratification for patients who survive an ICU stay. The follow up will include a careful tracking of patients through telephone calls and questionnaires at 3, 6 and 12 months after ICU discharge. FROG-ICU aims to identify the clinical and biological phenotype of patients with different levels of probability of death in the year after ICU discharge.

**Discussion:**

FROG-ICU has been designed to better understand long term outcome after ICU discharge as well as risk factors for all-cause and cardiovascular morbidity and associated mortality. It is a large prospective multicenter cohort with a biological (on plasma and urine) collection and one-year follow-up of ICU patients. FROG ICU will allow performing a risk stratification of ICU survivors as to recognize the subset of patients who may benefit from an early intervention to allow decreased cardiovascular morbidity and related mortality.

**Trial registration:**

ClinicalTrials.gov NCT01367093.

## Background

Numerous studies have been designed to investigate the early mortality of patients admitted in ICU. They focus on mortality at ICU discharge, at 28 days or/and at hospital discharge [1–3]. Case fatality is high, ranging from 14 to 44 % [1, 4–6] and up to 50 % in septic shock patients [7, 8]. Although many tools are available to assess the severity of illness and help clinicians to determine a prognosis during an ICU stay (Table 1), much less is known about what happens after ICU and hospital discharge.

**Table 1:** The severity of disease classification systems (or risk scores) most frequently used to evaluate the severity of ICU patients

Score	Patients	Endpoint	Measure	Reference
Acute Physiolosy and Chronic Health Evaluation (APACHE)	All ICU patients	Hospital mortality	Within 24 h of ICU admission	Knauss WA, 1981, 1985 and 1991[67–69], Zimmerman JE 2006 [70]
Simplified Acute Physiology Score (SAPS)	All ICU patients	28-day mortality	Once, at 24 h after ICU admission	Le Gall JR, 1993 [50] and Moreno RP 2008 [71]
Sequential Organ Failure Assessment (SOFA)	All ICU patients	ICU mortality	Daily	Vincent JL, 1996 [51]
Multiple Organ Dysfunction (MOD) score	All ICU patients	ICU and hospital mortality	Daily	Marshall J, 1995 [72]
McCabe Classification	Infective patients	Hospital mortality	At admission	McCabe WR, 1962 [52]
Sabadell Score	All ICU patients	Hospital mortality	At discharge	Fernandez R, 2006 [73]
Glasgow Coma Scale (GCS)	Traumatic brain injury	Quantify level of consciousness	Daily	Teasdale GM, 1974 [53]
Confusion Assessment method for the ICU CAM-ICU)	All ICU patients	Identify delirium	Daily	Ely EW, 2001 [54]
Injury Severity Score (ISS)	Trauma patients	Hospital mortality	At admission	Baker SP, 1974
Trauma Score	Trauma patients	Hospital mortality	At admission	Champion HR, 1981
Lung Injury Score (Murray Score)	Lung injured patients	Quantify the severity of lung injury	Daily	Murray JF, 1988

To reduce the mortality rate of ICU survivors, it is important to identify the group of patients who have a higher probability of death in the year following ICU discharge and to recognize the adjustable factors associated with mortality. Few data have been published regarding the long-term outcome of ICU patients, and most importantly, there are no recommendations for the long-term management of these patients, only experts opinion have been published [9, 10]. Some studies have demonstrated that mortality rates among ICU survivors are higher compared to the general population [11–15] and that ICU stay impact on patients’ quality of life [16]. Moreover, other studies [14, 15] found that this over-risk of mortality rates is sustained over time persisting after 5 or 15 years of follow-up. Three studies [11–13] reported a worse survival rate for the ICU patients than that for the age-matched control population in the first year after ICU discharge, they also observed that after 2 to 4 years, the survival curves of the two groups became parallel.

Table 2 describes the 15 largest studies from the last 30 years, investigating the one-year outcome of ICU patients and their associated risk factors [11, 13, 15, 17–28]. Using these 15 studies, the pooled estimate of post-ICU one-year mortality rate was 18.8 % (95 % confidence interval: 15.1 – 22.5 %), and the corresponding overall mortality (ICU + post-ICU one-year mortality) was 33.4 % (95 % CI: 27.0 – 39.9 %). However, only six papers out of fifteen had a prospective and multicenter study design (most were post-hoc analyses of prospectively collected data) [11, 19, 20, 23–25]. As expected older age was always associated with long-term mortality, along with APACHE score at admission (or within the first 24 h of the ICU stay). Other factors associated with long-term mortality were the presence and type of comorbidities and the cause of ICU admission.

**Table 2:** The 15 largest studies including consecutive ICU patients, reporting one-year outcome and published in the last 30 years

Name	Country	Study design	Recruitment period	Sample size	Age (years)	Sex (M %)	Severity score	ICU mortality	One-year mortality after ICU discharge	Overall mortality	Factors associated with one-year mortality
Zaren B, 1989 [12]	Sweden	O/P/S	1983	978	53.6	58	N/R	9.6 %	18.7 %	26.5 %	age, AC (cardiac arrest, MOF, neurological or CV disease), chronic condition (DM, CHF, cortisone medication)
Dragsted L, 1989 [13]	Denmark	O/P/S	1979-1983	1308	60	50.6	N/R	18.3 %	29.8 %	42.7 %	Age, gender, medical admission category, cancer
Rochwood K 1993 [14]	Canada	O/P/M	N/R	884	N/R	63.2	18.8 .(APACHE II)	14.4 %	23.5 %	34.5	Age
Niskanen M, 1996 [53]	Finland	O/P/M	1987	12180	57.2	62.9	11.7 (APACHE II)	9.9 %	20 %	27.9 %	Age, gender, emergency admission, APACHE II at admission, cancer, CV dz, RF, GI dz, cardiac arrest
Douglas C, 2002 [15]	USA	O/P/M	Feb 1997 - Mar 1999	538	65.8	56.3	N/R	47.4%^a^	32.5 %	64.5 %	N/R
Keenan SP, 2002 [16]	Canada	O/R/M	Apr 1994- Mar 1996	27103	54.3	57.1	N/R	14.3%^a^	10.9 %^b^	N/R	Age, comorbidity (lymphoma/leukemia, HIV, RF)
Kaarlola A, 2003 [17]	Finland	O/P/S	1995	591	N/R	N/R	N/R	N/R	N/R	36 %	N/R
Wright JC, 2003 [9]	UK	O/R/S	Jul 1985- Jul 1992	2104	53.6	N/R	14 (APACHE II)	20.6	20.2	36.7 %	Age, APACHE II, AC(hematological and neurological dz, septic shock)
Bagshaw SM, 2006 [18]	Canada	O/P/M	May 1999 - May 2002	5693	64.9	62	24.9 (APACHE II)	13.4 %	12.9 %	24.5 %	Age, medical diagnosis at admission, APACHE II score, AKI
Williams TA 2008 [11]	Australia	O/P/S	1987-2002	22298	61	67	11 (APACHE II)	10.7%^a^	5.4 %	15.5 %	Age, comorbidity, AC, new diagnosted cancer
Orwelius L, 2010 [19]	Sweden	O/P/M	Aug 2000- Jun 2004	2586	N/R	N/R	N/R	10.2 %	24 %	31.4 %	N/R
Braun A, 2012 [20]	USA	O/P/M	Nov 1997 - Apr 2009	51815	61.7	58.2	N/R	13%^a^	15.3 %	26.3 %	Low preadmission 25(OH)D level
Meynaar IA, 2012 [21]	Holland	O/R/S	Jan 2004 – Dec 2009	3477	N/R	N/R	N/R	8.2 %	20.1 %	26.7 %	Age, APACHE II, discharge not toward home
Grander W, 2013 [22]	Austria	O/P/S	Jan 2001- Jun 2004	1086	N/R	N/R	N/R	9.3 %	15.7 %^b^	N/R	HR before ICU discharge
Luangasanatip N, 2013 [23]	Thailand	O/R/S	Jan 2004 - Dec 2005	10321	57.6	N/R	N/R	31.5 %	20.7 %	45.7 %	AC (cerebrovascular dz, cancer)
FROG ICU	Europe	O/P/M	From Apr 2011 to Dec 2013	2250 (expected)							

*ICU* intensive care unit, *O* observational, *R* retrospective, *P* prospective, *S* single-center, *M* multi-center, *N/R* not reported, *CPR* cardiopulmonary resuscitation, *AKI* acute kidney injury, *WBCC* white blood cells count, *AC* admission category, *MOF* multi organ failure, *DM* diabetes mellitus, *CHF* chronic heart failure, *dz* disease, *CV* cardiovascular, *RF* respiratory failure, *GI* gastrointestinal, *25(OH)D* 25-idrossicolecalciferolo, *HR* heart rate

^a^Hospital mortality (ICU mortality was not available)

^b^Mortality rate was extrapolated from a Kaplan-Meier curve

The association between some biomarker plasma levels and ICU mortality has been studied previously. Although it has been shown that troponin is frequently elevated in ICU patients (from 42.1 to 47.3 %) [29, 30], only 22.2–25.8 % of those patients meet the diagnostic criteria for myocardial infarction. More importantly, elevated cTn during ICU stay was associated with an increased risk of ICU and hospital mortality (OR 2.53; 95 % CI, 1.89 to 3.38) [31–36]. Natriuretic peptide (NPs) levels were also associated with patients’ outcome. It has been demonstrated that brain natriuretic peptide (BNP) level can predict mortality at 6 months after hospital discharge for patients admitted with acute heart failure [37]. These results have been confirmed by other investigators in heart failure patients admitted to the ICU [38]. High BNP levels are also associated with 30-day mortality in septic shock patients [39, 40]. Similarly, biomarker of inflammation and renal function have been shown to be associated with mid- and long-term outcome [41–47].

Thus, measuring cardiovascular, inflammatory and/or renal biomarkers at ICU discharge could be useful in early recognition of high risk patients in whom potential preventive or curative strategies could improve outcomes. Furthermore measuring biomarkers at discharge may allow risk stratification of patients for whom close clinical monitoring as well as chronic life-saving treatments should be introduced. For instance, implementation of oral beta-blocker at ICU discharge has proven benefits in acute heart failure patients who were admitted to the ICU for inotropic treatment (dobutamine or levosimendan) and who received oral beta-blocker therapy at discharge [48]. This has been confirmed on survival at one year after discharge in ICU patients admitted for acute respiratory failure [49].

Here, we describe the design of the French and euRopean Outcome reGistry in Intensive Care Unit (FROG-ICU), the main objective of which is to assess the incidence and identify the risk factors of mortality during the year following discharge from the ICU. This identification of the risks will be based on the patients’ evaluations throughout their ICU stays, using clinical and “routine” biological parameters (including creatinine, sodium, potassium, hemoglobin, etc.…) as well as ICU severity score systems that are already being used to assess short-term mortality as described in Table 1. In addition, the effectiveness of novel cardiovascular biomarkers, including highly sensitive troponin, natriuretic peptides, sST2 and adrenomedullin (Table 3) to early assess adjustable factors associated with long term mortality will be evaluated.

**Table 3:** Cardiovascular, inflammatory and renal biomarkers of potential interest in predicting long-term mortality in critically ill patients

Name	Function	Clinical interest	References
Cardio-vascular biomarkers			
copeptine	Peptide of stress deriving from vasopressine	marker of cardiovascular disease	Khan SQ, 2007
Proenkephalin	endogenous opiod polypeptide hormon	marker of cardiovascular and cerebrovascular disease	Seizinger BR, 1985
Troponin I us	part of troponin complex, heart contraction	marker of myocyte injury	Labugger R, 2000
Troponine T hs	part of troponin complex, heart contraction	marker of myocyte injury	Labugger R, 2000
Brian natriuretic peptide (BNP)	increase of natriuresis and decrease of vasculare resistance	marker of myocyte stress	Davidson NC, 1994
N-terminal pro-BNP (NT-proBNP)	biologically inactive segment of BNP	marker of myocyte stress	Moe GW, 2007
adrenomedullin (ADM)	vasodilatation, induction of angiogenesis, protection against oxydative stress and hypoxic injury	marker of myocyte stress	Khan SQ, 2007
Soluble ST2	involved in cardiac remodeling and fibrosis	marker of myocyte stress	Shah RV, 2010
galectine 3	involved in inflammation, fibrosis and neoplastic transformation	marker of heart failure	de Boer RA, 2009
Biomarkers of infection and/or inflammation			
C-reactive protrine (CRP)	acute-phase protein	marker of inflammation/infection	Elster SK, 1956
Interleukine 6	pro-inflammation and anti-inflammation cytokine	marker of inflammation/infection	Castell JV, 1990
procalcitonin (PCT)	precursor of calcitonin	marker of infection, mostly bacterial	Jones AE, 2007
Renal biomarkers			
Plasmatic cystatin C	protein derived by all nucleated cells, readsorbed by proximal tubular cells	marker of decrease glomerular filtration rate	Roos JF, 2007
Urinary cystatin C	protein derived by all nucleated cells, readsorbed by proximal tubular cells	marker of renal tubular injury	Roos JF, 2007
Plasmatic and urinary neutrophil gelatinase associated lipocain (NGAL)	involve in innate immunity	marker of renal tubular injury	Kjeldsen L 1993

## Methods and Design

### Study design

**Fig. 1:**
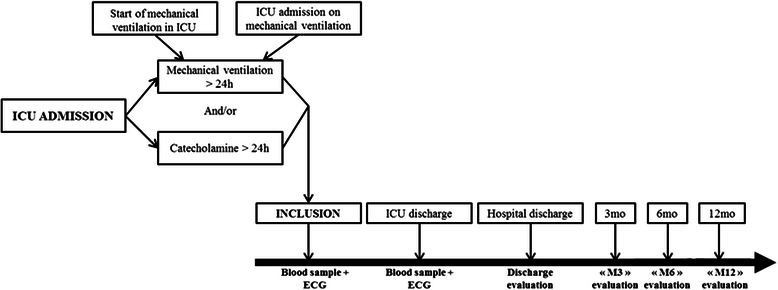
FROG-ICU study schema

The French and European Outcome Registry in Intensive Care Units (FROG ICU) study is a prospective, observational, multicenter cohort study, designed to assess the incidence and to identify the risk factors of mortality during the year following discharge from the ICU. The study will be conducted in France and in Belgium in accordance with Good Clinical Practice, Declaration of Helsinki 2002, validated by the ethical committee (Comité de Protection des Personnes - Ile de France IV, IRB n°00003835. Comission d’éthique biomédicale hospitalo-facultaire de l’hôpital de Louvain, IRB n°B403201213352) and was registed on ClinicalTrials.gov (NCT01367093). The study consists of two phases (Fig. 1): initially, all patients admitted to any of the participating centers during the recruitment period will be screened for the eligibility criteria listed below; subsequently, all patients included in the study who survive to ICU discharge will be followed up for one year through a telephone call and postal questionnaires at 3, 6 and 12 months.

### Participants

The study will involve 28 medical, surgical or mixed ICUs in 19 hospitals. The study cohort will include all consecutive patients who are admitted in ICU to any of the participating centers during the recruitment period when the following inclusion criteria are met: invasive mechanical ventilation support for at least 24 h and/or treatment with a positive inotropic agent (except dopamine) for more than 24 h. Because the ethical committee waived the need for written consent, all patients and/or next of kin will be informed and will orally consent to participate; consent was documented in the medical record by the investigator. Exclusion criteria are the following: less than 18 years old; severe head injury (initial Glasgow Coma Scale < 8) or brain death or a persistent vegetative state; pregnancy or breastfeeding; transplantation in the past 12 months; not expected to survive or to leave the hospital; and/or no social security coverage.

### Duration of the study and number of participating centers

The duration of participation for a patient will be a maximum of 12 months after ICU discharge. The inclusion period for any center is 32 months, and we hypothesize that the average number of patients to be recruited in each center would be 10 per month. The total study duration is 44 months.

### Study objectives

#### Primary objective

The primary purpose of this study is to assess the incidence of all-cause mortality in the year following an ICU stay and to identify the factors associated with mortality; those factors include both the clinical-biological factors and the ICU risk scores.

#### Secondary objectives

The secondary objectives of this study are: 1) to determine the incidence and the risk factors of cardiovascular morbidity and mortality in the year following an ICU stay; 2) to evaluate quality of life in the year following an ICU stay; 3) to assess the role of novel biomarkers in assessing long-term outcome after ICU discharge (Table 3).

### Data collection and biological samples

For each patient included, an electronic case report form will be completed that documents relevant information about the ICU stay and the one-year follow-up period. At the time of inclusion, the following will be collected: demographics, data on past medical history, ICU admitting diagnosis, hemodynamic (non-invasive and invasive) and respiratory parameters, severity of disease classification systems (SAPS-II, SOFA, CAM-ICU, McCabe Classification and GCS) [50–54]; digital electrocardiogram (EKG), recorded with CarTouch (Cardionics S.A., Bruxelles, Belgium), a high-definition electrocardiograph provided with TeleTouch software for the automatic transfer of the EKG to the informatics system. Routine laboratory tests will be performed. In 9 ICUs, an echocardiogram will also be performed within a few hours of inclusion. During the first 3 days of each ICU stay, respiratory and cardiovascular parameters (including hemodynamic and EKG) will be recorded daily and subsequently twice a week; SOFA score will be performed the first 3 days after inclusion, and routine biomarkers will be measured according to physician practice. Renal replacement therapy, death and cause of death in ICU will be collected.

The following data will be collected at ICU discharge: clinical parameters, digital EKG and routine biological assessment, ICU length of stay, duration of mechanical ventilation, SOFA score, treatment and results of echocardiogram at discharge when performed.

All patients included in the study population who survive to ICU discharge will be followed up for one additional year or until death, except for severely disabled subjects (Glasgow Outcome Scale < 4 at discharge). One-year outcome will be evaluated through data collection at 3, 6 and 12 months after ICU discharge. At those time points, patients will be contacted by telephone, and information about vital status (patient dead or alive), re-hospitalization, and cardiovascular morbidity will be recorded. For patients lost of follow-up, the vital status will eventually be checked through the national health services records when available. In addition, 4 follow-up questionnaires will be submitted to the study population: the Hospitalization Anxiety and Depression Scale (HADS) [55] and the Short Form-36 (SF-36) [56, 57] at discharge and at 3, 6, and 12 months after and the Impact of Event Scale-Revised (IES-R) [58] at 3, 6 and 12 months; a social questionnaire for assessing patients’ social environments (actual occupation, resumption of activity, level of income, marital status,…) will be submitted at 3 months after discharge.

A biological collection will be created with blood (3x10 ml in EDTA and aprotinin) and urine (10 ml) samples collected at two different time points: admission and discharge. The biological collection will allow for the measurement of novel biomarkers in a central laboratory. Table 3 lists the biomarkers with potential interest for the risk stratification of patient who survive an ICU stay.

### Statistical analysis

Primary analysis will be performed on all patients included in the cohort. Only patients who withdrew consent will be excluded. Patients who decided to stop tracking or who were lost to follow-up will be included in the analysis. Any missing value will be replaced by the previous value (the last value carried forward method). Sensitivity analyses for missing values will be conducted, for example, using a multiple imputation (MICE, multiple imputation by chained equations [59]. All tests will be bilateral formulation, with a risk of type I error of 5 %.

Calculating the number of subjects required is based on the primary endpoint, i.e., the impact and the risk factors on one year all-cause mortality. The study of the literature and the completion of the preliminary inquiry (conducted in December 2009) in 14 centers that participated to the study conduct us to assume a 1-year mortality after ICU discharge of 18 %. To ensure detection with a power of 80 % of binary prognostic factors with a prevalence of 33 % and an expected OR of 1.5 and a probability of death of approximately 18 %, 1636 patients should be included in this study [60]. Assuming a rate of refusal and/or follow-up losses of 10 %, the number of patients to be included in the one-year follow-up amounts to 1800. The expected in-ICU mortality rate is 25 %. Thus, taking into account these results, the number of patients to be included is 2250.

The primary analysis will be based on the research of the determinants of 1-year mortality of patients included in the FROG-ICU (i.e., ICU patients who needed mechanical ventilation for more than 24 h and/or treatment with a positive inotropic agent over 24 and who were discharged alive from the ICU) among clinical and biological variables and biomarkers of common interest (i.e., troponins, natriuretic peptides). The regression model used will be the logistic model. The log-linearity of the quantitative variables will be systematically evaluated, and, if appropriate, variable transformations will be performed. A selection model process will be performed using stepwise selection method. The selection process model will be further validated by bootstrap [61]. The existence of any collinearities will be observed, and a test of goodness of fit will be performed using the test and the van Cessie Houwelingen method [62]. Finally a cross-validation procedure will be performed. The measures of association will be provided, with odds ratios and confidence intervals at 95 %. An estimate of related risks will also be conducted. Finally, the overall incidence of 1-year mortality will be estimated at a confidence interval of 95 %.

As it is now recognized that highlighting the statistically significant association between new biomarkers and patient outcomes is not sufficient to demonstrate the interest of these biomarkers in terms of risk prediction [63–65]. We will use the proposed methodology of Pencina et al., which has been used in multiple articles of application. For each biomarker of interest, the net reclassification improvement (NRI) and integrated discrimination improvement (IDI) will be calculated, and comparisons between different biomarkers will be performed [64].

Regarding quality of life, a strategy similar to that outlined for the 1-year mortality analysis will be used to assess its impact and to highlight the predictors of impaired QoL at 6 months or 1 year. QoL will be estimated using the SF-36 score (ranging from 0 to 100) [66]. Altered QoL is considered when SF-36 is lower than 50. In addition, the value of the SF-36 measured in the FROG ICU study will be compared with the values that are typically found in healthy populations of the same age and sex. Interaction between quality of life and social life will be assessed by using information from the social questionnaire.

Last, regarding stress and anxiety, as previously described, IES-R higher than 30 will define post-traumatic stress disorder [58] and HADS higher than 8 will defined anxiety [55]. Similarly, analyses will consist in seeking to identify risk factors of stress and anxiety after ICU discharge.

## Discussion

Very limited information is currently available concerning the risk factors for long-term survival after a stay in the ICU. Some studies have investigated the long-term prognosis of ICU patients [11, 13, 15, 17–28], but only a few of them have been properly designed to achieve this result, in a prospective, multicenter manner and using a large number of patients [11, 19, 20, 23–25]. Few studies have evaluated biological parameters [25–27] as factors associated with long-term mortality, and only one study has correlated the patient’s condition at discharge with the long-term risk of death [27]. Consequently, at the moment, there are neither guidelines nor recommendations to assist clinicians in providing optimal patient management after ICU.

Accordingly, FROG-ICU is an innovative study that reflects the endeavor of many ICU physicians to assess the incidence of all-cause mortality in the year after ICU discharge and to identify the factors associated with this mortality. The strengths of the present study are listed in Table 4: first is the study design because FROG-ICU is a large observational, prospective and multicenter cohort study; second, the evaluation of each patient is performed through a comprehensive clinical assessment, instrumental tests (electrocardiogram, echocardiogram) and biological parameters; finally, all of the data are collected at admission, during the stay and at discharge.

**Table 4:** Strengths of the present study

Strengths of FROG-ICU study	
Study design	Observational, non-interventional, prospective and multicenter cohort study
Methods for the evaluation of the patient	Complete clinical assessment, instrumental tests (electrocardiogram, echocardiogram), biological parameters and routine biomarkers during ICU stay
Constitution of a biobank	For each patient, plasma and urine will be collected. For ICU-survivor, urine and plasma at discharge will also be collected
Repeated evaluations of the patient	At admission, during the ICU stay, at discharge and during one year

Preliminary studies suggest that pre-discharge therapy, especially the oral administration of beta-blockers, may improve the survival after an ICU stay [48, 49]. This would be confirm or not using the FROG-ICU data notably by using causal inference approaches. Moreover all of these findings favor developing a clinical trial with inclusion criteria and number of patients to be recruited based on the present study, with the main purpose of decreasing long-term all-cause and particularly cardiovascular morbidity and mortality after ICU discharge.

To sum up, FROG-ICU has been designed to better understand long term outcome after ICU discharge as well as risk factors for all-cause and cardiovascular morbidity and associated mortality. Because it is a large prospective multicenter study that collects clinical, biological and focused cardiovascular biomarkers, FROG ICU will allow to perform a risk stratification of ICU survivors as to recognize the subset of patients who may benefit from an early intervention to allow decreased cardiovascular morbidity and related mortality. Should the cardiovascular risks be identified here and validated in a sub-cohort, a randomized controlled trial using the results of this risk stratification will be in order to demonstrate survival benefits from a pre- and post-discharge multifaceted intervention that will include close cardiovascular monitoring and oral cardio-vascular therapies.
